# 80例复合型小细胞肺癌的临床分析

**DOI:** 10.3779/j.issn.1009-3419.2015.03.06

**Published:** 2015-03-20

**Authors:** 扬 罗, 周光 惠, 琳 杨, 峻岭 李

**Affiliations:** 1 100021 北京，中国医学科学院肿瘤医院肿瘤内科 Department of Medical Oncology, Cancer Hospital (Institute), Chinese Academy of Medical Sciences & Peking Union Medical College, Beijing 100021, China; 2 100021 北京，中国医学科学院肿瘤医院放疗科 Department of Radiation Oncology, Cancer Hospital (Institute), Chinese Academy of Medical Sciences & Peking Union Medical College, Beijing 100021, China; 3 100021 北京，中国医学科学院肿瘤医院病理科 Department of Pathology, Cancer Hospital (Institute), Chinese Academy of Medical Sciences & Peking Union Medical College, Beijing 100021, China

**Keywords:** 肺肿瘤, 病理, 预后, Lung neosplasms, Pathology, Prognosis

## Abstract

**背景与目的:**

复合型小细胞肺癌（combined small cell lung cancer, C-SCLC）的检出率逐年增高，但其相关报道仍较少，本研究探讨C-SCLC的临床病理特征与预后的关系，分析其治疗现状。

**方法:**

回顾性分析2006年1月-2011年12月间80例病理证实的C-SCLC患者的资料，采用*Kaplan*-*Meier*法计算生存率，*Log*-*rank*法进行单因素预后分析，*Cox*风险回归模型分析影响总生存（overall survival, OS）的因素。

**结果:**

全组的OS为0.3个月-81.4个月，中位OS为26.2个月。单因素分析显示：性别、治疗前卡氏评分、肿瘤直径、分期是影响OS的预后因素（*P*＜0.05）。多因素分析显示，只有TNM分期是独立的影响OS的因素（*P*=0.015）。全组多数患者（75.0%）采取综合模式治疗，以铂类药物为基础的联合化疗是主要的治疗方法，采用SCLC的化疗方案或非小细胞肺癌的方案对患者生存的影响无统计学意义（*P*＞0.05）。

**结论:**

C-SCLC是一种特殊类型的肺混合性癌，治疗应采用以铂类为基础的化疗为主的综合治疗模式，TNM分期是独立的预后影响因素。

## Abstract (en)

**Background and objective:**

Studies on combined small cell lung cancer (C-SCLC), which shows continuously increasing incidence, are rare. Accordingly, we explored the relationship between the clinicopathological characteristics and prognostic factors of C-SCLC patients and analyzed the treatment of this disease.

**Methods:**

Between January 2006 and December 2011, 80 patients with pathologically confirmed C-SCLC were retrospectively analyzed. The *Kaplan*-*Meier* methods were used to calculate the survival rate, and the *Log*-*rank* test was used to examine differences between arms. The Cox regression model was used to analyze the independent factors affecting the overall survival (OS).

**Results:**

The OS of the C-SCLC patients in all groups had a median of 26.2 mo and a range of 0.3 to 81.4 mo. In univariate analysis, gender, Karnofsky performance score before treatment, tumor diameter, and tumor stage were the considered prognostic factors affecting the OS rate of the C-SCLC patients (*P* < 0.05). *Cox* multivariate analysis showed that only the TNM stage was the independent prognostic factor influencing the OS of the C-SCLC patients. The majority (75.0%) of the patients received multimodality therapy and platinum-based chemotherapy as the main treatment. However, no significant differences in OS rate were observed between patient groups under conventional SCLC regimens and non-SCLC regimens (*P* > 0.05).

**Conclusion:**

C-SCLC is a specific mixed carcinoma. Combined therapy with platinum-based chemotherapy as the main treatment should be adopted in the therapeutic regimen of this disease. TNM stage was the independent prognostic factor influencing the OS of C-SCLC patients.

复合型小细胞肺癌（combined small cell lung cancer, C-SCLC）是一类由SCLC与非小细胞肺癌（non-small cell lung cancer, NSCLC）成分相混合的癌，其中NSCLC成分可以是鳞癌、腺癌、大细胞癌，甚至是少见的梭形细胞癌、巨细胞癌、肉瘤样癌等，且混合的NSCLC病理成分可以为一种或多种^[1-3]^。既往报道^[4, 5]^C-SCLC的发病率与诊断率较低，仅占全部SCLC的1%-3%，近年来随着诊断技术的不断提高，其检出率有逐步增高的趋势。但迄今为止，针对该病的相关报道仍较少。因此，我们开展了回顾性研究，结合文献探讨C-SCLC的临床病理特征及其与预后的关系，分析其治疗现状，旨在提高对该病诊断和治疗上的认识。

## 1 资料与方法

### 1.1 一般临床资料

2006年1月-2011年12月在中国医学科学院肿瘤医院治疗的C-SCLC患者共80例，患者的临床资料详见表 1，全部诊断均经组织病理学证实，分期检查包括体格检查、胸部计算机断层扫描（computed tomography, CT）、正电子发射计算机断层显像（positron emission CT, PET-CT）、颈腹部超声、脑磁共振成像（magnetic resonance imaging, MRI）和骨扫描等，采用2009年美国癌症联合委员会（American Joint Committe on Cancer, AJCC）第7版TNM分期及美国退伍军人肺癌协会（Veterans Administration Lung Study Group, VALSG）分期^[6]^（局限期指肿瘤位于一侧胸腔，即使发生了局部转移和同侧锁骨上淋巴结转移，只要它们与原发灶能覆盖于同一放射野，也属于局限期，没有胸腔外转移也被称为局限期，除此之外属于广泛期）。

**1:** 80例复合型小细胞肺癌患者一般临床资料 The characteristics of the 80 patients with C-SCLC

Characteristics	*n*	Proportion%
Age (yr)		
≤60	48	60.0
> 60	32	40.0
Gender		
Male	63	78.8
Female	17	21.3
Smoking history		
Yes	62	77.5
No	18	22.5
Karnofsky score		
≥80	71	88.8
< 80	9	11.3
Weight loss		
Yes	13	16.2
No	67	83.8
X-ray typing		
Central	66	82.5
Peripheral	14	17.5
VALSG staging		
Limited disease	56	70.0
Extensive disease	24	30.0
AJCC 7^th^ stage		
Ⅰ	8	10.0
Ⅱ	13	16.3
Ⅱ	37	46.3
Ⅳ	22	27.5
Cell mixture components		
Squamous cell carcinoma	35	43.8
Adenocarcinoma	24	30.0
Large cell carcinoma	10	12.5
Atypical carcinoid	1	1.3
Others	10	12.5
Treatment modes		
Single-mode	20	25.0
S+R	1	1.3
S+C	24	30.0
R+C	21	26.3
S+C+R	14	17.5
Chemotherapy regimens^*^		
Regimens for SCLC	47	66.2
Regimens for NSCLC	19	28.8
^*^71 patients received chemotherapy with regimens unknown in 5 patients. S: surgery; R: radiotherapy; C: chemotherapy; AJCC: American Joint Committe on Cancer; VALSG: Veterans Administration Lung Study Group; NSCLC: non-small cell lung cancer.		

### 1.2 组织标本来源

包括原发灶手术切除标本46例、支气管镜活检15例、肺穿刺活检4例、转移淋巴结活检3例、其他转移部位活检2例、痰细胞学和支气管镜活检共同诊断6例、痰细胞学和肺穿刺病理共同诊断2例、痰细胞学和转移淋巴结穿刺病理共同诊断1例、支气管镜和转移淋巴结穿刺共同诊断1例。46例术后病理诊断的患者中，21例在术前未能正确诊断为C-SCLC，包括13例支气管镜活检病理，5例痰或支气管镜刷片细胞学，3例肺穿刺病理。

### 1.3 研究因素与观察指标

研究因素包括年龄、性别、吸烟史、卡氏评分、体重是否减轻、肿瘤大小、肿瘤位置、TNM分期、VALSG分期、病理混合成分等。不吸烟定义为吸烟＜100支，体重减轻定义为半年内体重下降＞5%。总生存（overall survival, OS）定义为从首次治疗开始至死亡或末次随访的时间。无病生存（disease-free survival, DFS）：接受根治手术的患者从手术治疗开始至肿瘤复发转移、死亡或末次随访的时间。

### 1.4 统计学方法

随访截至2013年12月31日，6例患者失访，随访率为92.5%。采用SPSS 20.0软件行*Kaplan*-*Meier*法生存分析，*Log*-*rank*法单因素预后分析，*Cox*风险回归模型进行多因素分析。*P*＜0.05为差异有统计学意义。

## 2 结果

### 2.1 临床病理特点

全组患者的中位年龄为59岁（范围20岁-79岁），其中男性63例（78.8%），女性17例（21.3%），62例（77.5%）患者有吸烟史，71例（88.8%）患者初诊时卡氏评分≥80分，C-SCLC以中央型居多（66例，82.5%）；局限期较广泛期多见（2.3:1）；采用AJCC第7版，Ⅰ期、Ⅱ期、Ⅲ期和Ⅳ期的患者分别为8例（10.0%）、13例（16.3%）、37例（46.3%）和22例（27.5%）；病理混合成分以鳞癌最多见（43.8%），其他还包括腺癌、大细胞癌、不典型类癌等，其中混合≥2种NSCLC成分的4例。

### 2.2 生存结果

随访截至2013年12月31日，失访6例，死亡45例，1访、3访、5年生存率分别为71.6%、59.6%、35.2%。全组的OS为0.3个月-81.4个月，中位OS为26.2个月。

### 2.3 手术患者的失败模式

46例手术治疗的患者中，22例死亡，其中术后并发症死亡2例，非肿瘤相关死亡1例，肿瘤相关死亡19例，中位OS为34.2个月；25例患者出现肿瘤复发转移或死亡，中位DFS为22.6个月，22例复发转移患者中17例患者有影像学诊断的肿瘤复发转移部位，包括单纯远处转移12例、单纯局部区域复发4例、1例同时出现局部区域复发和远处转移。

### 2.4 预后分析

单因素分析显示男性、术前卡氏评分＜80分、肿瘤直径＞3 cm、广泛期、Ⅲ期和Ⅳ期是OS的不良预后因素，而年龄、吸烟史、体重减轻、肿瘤X线分型、混合非鳞癌成分等因素未显示与预后相关（表 2）。将*P*＜0.05的5个变量进行*Cox*回归分析，只有TNM分期是独立的预后影响因素（*P*=0.015）（表 3）。Ⅰ期和Ⅱ期、Ⅲ期、Ⅳ期患者的5年OS率分别为47.6%，34.9%和11.5%（χ^2^=12.685, *P*=0.002）（图 1）。

**2:** 80例复合型小细胞肺癌患者的单因素预后分析 Single-factor prognostic analysis of 80 patients with C-SCLC

Clinical factors	*n*	1-yr survival rate (%)	3-yr survival rate (%)	5-yr survival rate (%)	*χ*^2^	*P*
Gender					4.526	0.033
Male	63	70.1	35.1	31.2		
Female	17	76.5	64.2	48.1		
Age (yr)					1.859	0.173
60	48	80.2	49.5	31.2		
> 60	32	62.5	30.7	24.5		
Weight loss					0.049	0.824
Yes	17	50.0	41.7	41.7		
No	63	75.5	40.9	33.1		
Smoking history					3.527	0.060
Yes	62	68.0	33.5	29.8		
No	18	83.3	66.2	53.0		
Karnofsky score					10.631	0.001
≥80	71	78.1	55.3	40.2		
< 80	9	22.2	0	0		
Tumor size					4.146	0.042
≤3 cm	24	63.2	57.9	57.9		
> 3 cm	56	63.1	34.1	26.5		
X-ray typing					0.323	0.570
Central	66	70.0	39.9	32.7		
Peripheral	14	78.6	57.1	47.6		
AJCC 7^th^ staging					12.685	0.002
Ⅰ+Ⅱ	21	95.0	72.7	47.6		
Ⅲ	37	64.1	34.9	34.9		
Ⅳ	22	62.4	23.0	11.5		
VALSG staging					6.029	0.014
Limited disease	56	76.0	48.6	44.2		
Extensive disease	24	61.3	25.5	12.8		
Cell mixture components					0.401	0.526
Squamous	35	69.8	48.8	40.7		
Non-squamous	45	72.9	36.6	32.0		

**3:** 预后因素的*Cox*回归分析 Multivariate prognostic analysis by *Cox* model

Factors	B	df	*P*	Exp(B)	95%CI for Exp(B)	
					Lower	Upper
VALSG staging	-0.879	1	0.154	0.415	0.124	1.389
Gender	0.578	1	0.055	1.783	0.988	3.218
Karnofsky score	0.685	1	0.127	1.984	0.824	4.779
Tumor size	0.494	1	0.204	1.638	0.765	3.508
TNM staging	1.050	1	0.015	2.857	1.223	6.677

**1:**
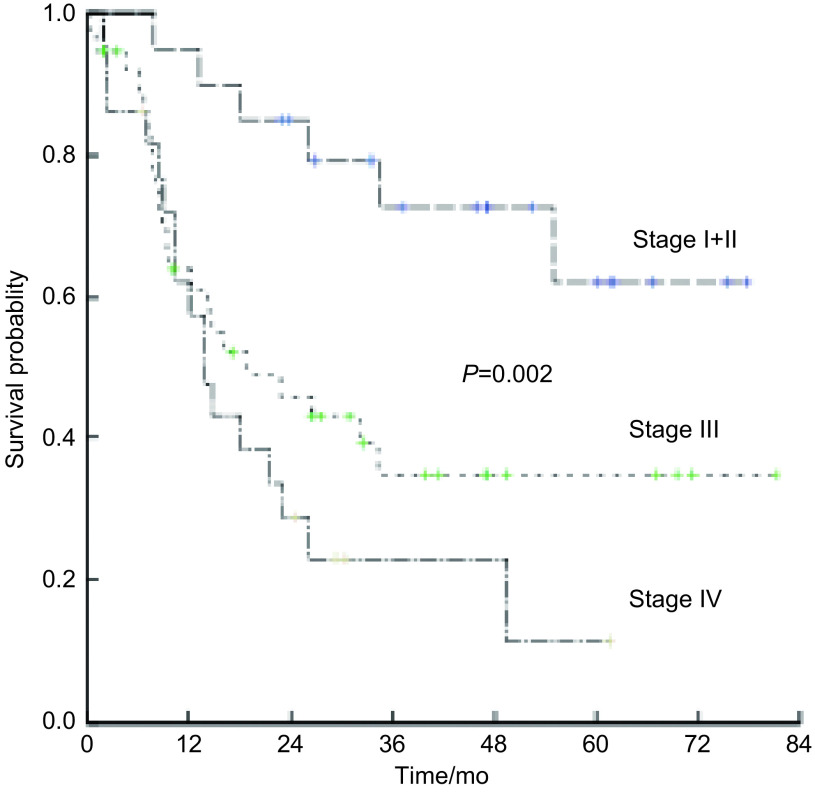
C-SCLC患者的生存曲线 Survival curve of the 80 patients with C-SCLC. C-SCLC: combined small cell lung cancer.

### 2.5 C-SCLC的治疗现状

全组中接受手术、化疗、放疗者分别为46例（57.5%）、71例（88.8%）和37例（46.3%）。大多数患者（60例，75.0%）接受了综合模式治疗，20例（25.0%）患者只接受了单一模式治疗（包括8例初诊为Ⅳ期只接受化疗；1例初诊为脑转移患者只接受放疗后死亡；1例初诊为Ⅱb期3例初诊为Ⅲ期患者化疗后进展未能接受局部治疗；2例患者术后并发症死亡；3例Ⅲa期患者术后短期出现转移；1例初诊为Ⅱb期和1例初诊为Ⅲa期患者术后随访未接受化放疗）。化疗是C-SCLC主要的治疗手段，71例接受化疗的患者中，5例方案不详，47例患者初次化疗采用SCLC常规方案，包括41例铂类联合足叶乙甙和6例顺铂联合伊立替康，19例初次化疗采用NSCLC常规方案（包括铂类联合紫杉类或吉西他滨或去甲长春花碱）。在术后患者的辅助化疗中，接受SCLC方案化疗和NSCLC方案化疗的3年生存率分别为61.0%和41.3%（χ^2^=1.177, *P*=0.278）；在Ⅳ期患者中，一线治疗接受SCLC方案化疗和NSCLC方案化疗的1年生存率分别为22.4%和28.6%（χ^2^=2.015, *P*=0.156），差异均无统计学意义。

## 3 讨论

C-SCLC的低发病率与诊断方法有关，Fraire等^[2]^的研究显示C-SCLC的检出率受活检标本的大小以及完整性、病理切片数目、以及是否手术或尸检结果的影响。与既往的细胞学诊断相比，支气管镜、肺穿刺、淋巴结活检等技术的进步使大多数患者可以取得病理组织学标本，诊断的完整性和准确率也相应的增加，但仍低于手术和尸检的结果^[4, 7]^。本组46例接受手术的患者中，21例（45.7%）患者术前的病理细胞学检查未能正确诊断为C-SCLC，包括13例支气管镜下病理，5例痰或支气管镜刷片细胞学，3例经皮肺穿刺病理，也证实了临床上C-SCLC患者的发病率被低估，尤其是在未接受手术治疗的患者。

C-SCLC好发于吸烟男性，中位年龄为58.0岁-66.4岁。病理结果显示C-SCLC中的NSCLC成分以鳞癌和腺癌为主。本研究的结果与文献报道相仿^[3, 8, 9]^，中位年龄59岁，78.8%为男性，77.5%的患者有吸烟史，混合NSCLC成分以鳞癌最多（43.8%），其次为腺癌（30.0%）。与单纯型SCLC相比，本组中C-SCLC患者的局限期病例占70%，其中早期（Ⅰ期-Ⅱ期）病例占全组的26%，远远高于单纯型SCLC文献^[10, 11]^报道的只有20%-25%的患者属局限期，仅10%的患者为早期（Ⅰ期-Ⅱ期）。其原因可能是C-SCLC的大部分病例经手术病理确诊，而手术患者多为早期病变，这属于病例选择上的偏倚。

关于C-SCLC的预后目前缺乏大样本的研究报道，吕旭等^[8]^回顾性分析44例C-SCLC患者，结果显示发生于右肺者预后优于发生于左肺者（*P*=0.001），中央型比周围型预后好（*P*=0.035）；复合病理NSCLC成分为大细胞癌者预后最差（*P*=0.031）；多因素生存分析显示病理混合成分及肿瘤位置为影响总生存的独立因素。罗洁等^[9]^总结了88例C-SCLC患者的临床资料，Ⅰ期和Ⅱ期、Ⅲ期、Ⅳ期患者的中位OS分别为39个月、10个月和7.8个月，差异有统计学意义（*P*=0.003）。本研究结果显示男性（*P*=0.033）、术前卡氏评分＜80分（*P*=0.001）、肿瘤直径＞3 cm（*P*=0.042）、TNM分期中Ⅲ期和Ⅳ期（*P*=0.002）和VALSG分期为广泛期（*P*=0.014）均是OS的不良预后因素。但是进一步的多因素分析结果显示只有TNM分期是独立的预后影响因素（*P*=0.015）。总的来说，由于每组报道的患者例数均较少并都是单中心回顾性分析，对患者的治疗缺乏统一标准，因此还需要大样本的研究或荟萃分析的结果进一步明确影响患者预后的因素。

针对C-SCLC治疗的文献报道很少，其治疗原则主要借鉴SCLC的治疗模式，强调综合治疗，但综合治疗模式仍不统一。Hage等^[4]^的结果显示，对于高度选择（外周型或临床分期为Ⅰ期）的C-SCLC患者，手术因其相对较高的治愈率而具有非常重要的价值，Ⅰ期C-SCLC患者手术后的5年生存率为31%，其中T1N0M0和T2N0M0患者的5年生存率分别为50%和25%，而对于临床分期为Ⅲ期的患者不建议手术，因为这部分患者不能从手术中获益，新辅助化疗和/或辅助化疗的价值仍无定论，但通常被认为可以减少远处转移的发生。而对于局部晚期的患者，门玉等^[12]^报道放疗可以显著提高局部晚期（Ⅲa期、Ⅲb期）（*P*=0.032）、淋巴结阳性（*P*=0.006）或术后阳性淋巴结＞4个（*P*=0.025）患者的OS。单纯型SCLC对化疗相当敏感，有效率高达60%-80%^[10]^，临床上，C-SCLC基本采用与单纯型SCLC相同的化疗方案，但是其对化疗的反应较单纯型SCLC差，有效率约为50%左右，导致C-SCLC对化疗疗效下降的原因可能是由于C-SCLC中混合的NSCLC成分对SCLC常规方案的敏感性较差^[13]^，因此，NSCLC的常规方案也被试用于C-SCLC的治疗中。本组的结果75.0%的患者（60例）接受了综合治疗，88.8%的患者接受化疗。在接受手术的患者中，失败仍以远处转移为主，提示需进一步研究更加有效的化疗方案。我们比较了41例接受SCLC常规方案和19例采用NSCLC常规方案患者的生存，无论是术后辅助治疗还是晚期初治患者，两组的生存相仿，差异无统计学意义。

综上所述，由于诊断需要充足的组织标本，所以C-SCLC的发病率存在低估；肿瘤的TNM分期是独立的预后不良因素；手术患者的失败仍以远处转移多见；目前推荐采用手术、放疗和化疗的综合治疗模式。本研究不足之处在于单中心回顾性分析，例数太少，可能存在选择偏倚，患者的化疗方案缺乏统一标准，期待有大样本的研究进一步探索。
